# Autoimmune pancreatitis

**DOI:** 10.1093/gastro/got011

**Published:** 2013-03-28

**Authors:** Gyanprakash A. Ketwaroo, Sunil Sheth

**Affiliations:** Department of Gastroenterology, Beth Israel Deaconess Medical Center, Harvard Medical School, Boston, MA, USA

**Keywords:** autoimmune pancreatitis, review

## Abstract

Autoimmune pancreatitis (AIP) is a rare, heterogeneous, fibroinflammatory disorder of the pancreas. It has gained increasing recognition due to a presentation that can mimic difficult-to-treat disorders such as pancreatic cancer, cholangiocarcinoma and primary sclerosing cholangitis. In contrast, autoimmune pancreatitis is a benign disease that is very responsive to therapy with corticosteroids.

There are two types of AIP. Type 1 disease is the most common worldwide and is associated with extrapancreatic manifestations and elevated levels of IgG4-positive cells. Type 2 AIP is characterized by a paucity of IgG4-positive cells and is more difficult to diagnose. This review provides an update on the diagnosis, pathophysiology and treatment of AIP, with special emphasis on the two subtypes.

## INTRODUCTION

Autoimmune pancreatitis (AIP) was first reported in 1961 in a case of pancreatitis in the setting of hypergammaglobulinemia [1]. In 1995, Yoshida *et al.* described a series of patients with features of chronic pancreatitis and simultaneous autoimmune characteristics, introducing the concept of autoimmune pancreatitis as a distinct entity [2]. Increasing awareness of AIP has led to recognition that it is a heterogeneous disorder with important variations in pathophysiology, genetic predisposition and extrapancreatic manifestations.

AIP can occur as a primary disease of the pancreas or as part of a systemic disease associated with elevations in levels of IgG4 producing cells [3, 4]. In this IgG4 systemic disease, other organs such as salivary glands, kidneys and bile ducts are involved. This has led to the classification of AIP into two subtypes. Type 1 AIP is characterized by a systemic IgG4-associated disorder with elevated IgG4-positive cells in serology, the pancreas and other organs. It is the most common form worldwide, accounting for almost all cases in Japan and Korea and more than 80% of cases in Europe and the United States [5, 6]. Type 2 AIP is primarily found in the pancreas, with a lack of IgG4-positive cells.

## EPIDEMIOLOGY

AIP is a rare disorder, with a reported prevalence in Japan of 0.82/100 000 [7]. The incidence of AIP in the United States is not fully known, with reports limited to case series and descriptions of tertiary referrals. In a Japanese nationwide survey of AIP, the peak age of onset was in the seventh decade, with 95% of patients older than 45 [7]. In an international study of 978 Type 1 AIP patients, the average age was 61.4 years at the time of diagnosis [6]. On the other hand, Type 2 patients were younger, with an average age of 39.9 years. In this study, the proportion of male patients was also significantly higher in Type 1 AIP than in Type 2 (77 vs 55%).

## CLINICAL PRESENTATION

Over time, as the pathophysiology of AIP and its clinical spectrum of disease have become clearer, it has been known by a variety of names. These include sclerosing pancreatitis, tumefactive pancreatitis and non-alcoholic destructive pancreatitis. It has been most recently known as IgG4-related disease.

The pancreatic disease in patients with AIP can manifest in a variety of ways including: (i) a focal mass or enlargement, which can be difficult to distinguish from pancreatic cancer [8]; (ii) strictures of the pancreatic duct and (iii) chronic or recurrent abdominal pain. Recurrent acute pancreatitis in the absence of biliary involvement is not a typical presentation of AIP. Likewise, significant weight loss and severe chronic abdominal pain are rare in AIP [9].

Patients can also present with manifestations due to other organ involvement, such as biliary disease, symptoms of Sjögren’s disease (due to salivary gland involvement), lung nodules, interstitial nephritis and retroperitoneal fibrosis, among others. Biliary tract involvement typically includes strictures, especially involving the proximal portions of the extrahepatic and intrahepatic ducts. There is an ever-expanding list of extrapancreatic manifestations as investigators discover new areas of IgG4 plasma cell infiltration [4].

Clinical presentation also varies depending on the subtype of AIP. In a recent international survey of 731 patients with AIP, the most common presenting symptom was obstructive jaundice [10]. This occurred in approximately 75% of patients with Type 1 AIP and 50% of patients with type 2 AIP [10]. However, patients with Type 2 AIP presented more commonly with acute pancreatitis (34%) and abdominal pain (68%) than patients with Type 1 AIP [10]. Likewise, while inflammatory bowel disease is associated with AIP, it is more commonly found in patients with Type 2 disease [10, 11].

## DIAGNOSIS

A variety of diagnostic scoring systems for AIP have been advocated around the world. In the United States, Chari *et al.* introduced the HISORt criteria based on a prospective study of 29 consecutive patients at the Mayo Clinic who met histological criteria for AIP [12]. These criteria were based on Diagnostic **H**istology, Characteristic **I**maging, Elevated serum IgG4 levels on **S**erologic testing, **O**ther organ involvement and **R**esponse to glucocorticoid **t**herapy. This was an update to the original Japanese Pancreas Society Criteria [13] and expanded on the histological and imaging characteristics of AIP, while including extrapancreatic manifestations and response to steroid therapy as diagnostic criteria. It remains one of the most commonly used diagnostic criteria in the United States.

In a small series of 26 patients with AIP at a large US center, application of the HISORt, Japanese Pancreas Society and Korean criteria diagnosed about 85% of patients [8]. In 2010, an international panel of experts developed consensus diagnostic criteria (ICDC) for AIP, which focused on the distinction between Type 1 and Type 2 AIP [9]. These criteria are based on the clinical profile of AIP, including characteristic histology and imaging, serum IgG4 levels, extrapancreatic manifestations and response to steroid treatment. For each criterion, there are two levels of evidence: typical or highly suggestive evidence (level 1) and indeterminate/suggestive evidence (level 2). With this stratification, Type 1 AIP can be confirmed with a variety of combinations of level 1 and level 2 evidence. For instance, dynamic CT/MRI showing typical AIP imaging of diffuse pancreatic enlargement with delayed enhancement and pancreatic ductal strictures without dilatation (level 1) and serum IgG4 level almost double the normal upper limit (level 2) would allow the diagnosis of Type 1 AIP to be made non-invasively [5, 9]. In contrast, by ICDC recommendations, definitive diagnosis of Type 2 AIP requires histology.

## HISTOLOGY

The traditional ‘gold standard’ for the diagnosis of autoimmune pancreatitis is characteristic histology. In patients with Type 1 AIP, the pancreas demonstrates a classic pattern known as lymphoplasmacytic sclerosing pancreatitis (LPSP). This is represented by periductal lymphoplasmacytic infiltration rich in IgG4, storiform fibrosis and obliterative venulitis.

In Type 2 AIP, the affected pancreas demonstrates neutrophilic infiltration in the ductal epithelium with duct destruction and occasionally microabscess formation [14, 15]. The histology is characterized by the classic ductal granulocyte epithelial lesion.

Tissue acquisition for histological diagnosis may be obtained by EUS with FNA or Tru-cut biopsy. Unlike FNA, which typically uses a 22-gauge needle to obtain aspirate for cytology, the large Tru-cut biopsy needle preserves tissue architecture, allowing for immunostaining and examination to diagnose AIP. Further, the larger needle acquires more material that may overcome some of the difficulty in diagnosis associated with the patchy distribution of the characteristic histological findings [16]. A recent review of the role of endoscopy in the diagnosis of autoimmune pancreatitis by Moon *et al.* highlighted that IgG4 immunostaining of pancreatic biopsies has a sensitivity of 11–88% and a specificity of 75–95% in diagnosing AIP [17]. Interestingly, Iwashita *et al.* have reported a sensitivity of 43% for EUS-FNA with a larger, 19-gauge needle in diagnosing AIP in a small series of 44 patients who met clinical criteria based on Japanese Pancreatic Society Guidelines [16]. In Type 2 AIP, where diagnosis can only be made definitively by histological findings, EUS-guided biopsies or large-needle FNA may be the least invasive methods of establishing the diagnosis.

## IMAGING

In clinical practice, AIP is often first diagnosed by radiologists who recognize the characteristic imaging findings on cross-sectional imaging with contrast-enhanced computed tomography (CT) or magnetic resonance imaging (MRI). On such cross-sectional imaging, a pancreas that is diffusely enlarged, with featureless borders and/or loss of lobular architecture, or ‘sausage-shaped’, is typical for AIP [18, 19]. The pancreas may also demonstrate delayed enhancement with or without rim-like enhancement on both MRI and CT [18, 19]. Peripancreatic stranding is usually minimal in AIP, unlike other forms of pancreatitis [18]. In 30–40% of cases of AIP, a focal pancreatic mass is found, which makes the distinction from pancreatic cancer difficult [20].

A long stricture of the pancreatic duct, without significant associated dilatation, is also highly characteristic of AIP [18, 21, 22]. This is best seen on endoscopic retrograde cholangiopancreatography (ERCP). A recent multicenter study highlighted four specific ERCP findings of AIP: (i) a long stricture (greater than one-third the length of the pancreatic duct), (ii) lack of upstream dilatation from the stricture, (iii) multiple strictures and (iv) side branches arising from the stricture site [17, 23]. Magnetic resonance cholangiopancreatography (MRCP) is a non-invasive but less accurate alternative to ERCP in evaluating these pancreatic ductal changes [24]. AIP may also manifest with narrowing of the intrapancreatic portion of the common bile duct, enhancing duct wall thickness and, stricturing and irregularity of the CBD.

EUS findings of AIP include diffuse hypoechoic pancreatic enlargement, bile duct wall thickening and peripancreatic hypoechoic margins [17, 25]. However, these findings are relatively non-specific and conventional EUS alone cannot be used to make the diagnosis. Alternatively, intraductal ultrasound can help to differentiate AIP from cholangiocarcinoma. On intraductal ultrasound, affected bile ducts associated with AIP have concentric wall thickening with smooth configuration and a smooth luminal surface [17] This is in contrast to the eccentric wall thickening and irregular luminal surface typical of cholangiocarcinoma.

## SEROLOGY

Given the presumed autoimmune etiology, there is an association of AIP with elevated levels of gammaglobulins and autoantibodies. In particular, IgG4-positive plasma cells may play a primary role in the pathogenesis. Serum IgG4 level >140 mg/dL has a sensitivity of 76% and specificity of 93% in diagnosing autoimmune pancreatitis, based on a Mayo Clinic cohort that included 45 AIP patients and 465 controls [26]. A meta-analysis of seven studies, evaluating the usefulness of serum IgG4 in diagnosing AIP, showed variation in sensitivity and specificity ranging from 67–94% and 89–100%, respectively [27]. Notably, 5% of healthy persons and 10% of patients with pancreatic CA have elevated IgG4 [28]. Consequently, elevated IgG4 antibodies alone cannot be used to make the diagnosis of AIP. Further, in Type 2 AIP, elevation in serum IgG4 is uncommon [11].

Antibodies to the peptide showing homology with an amino acid sequence of plasminogen-binding protein (anti-PBP) may have increased sensitivities of 93–95% for autoimmune pancreatitis [29]. These antibodies were found in patients who were serum IgG4-negative, suggesting that they may be useful for Type 2 AIP and the cohort of Type 1 AIP with negative serology. However, 5% of patients with pancreatic cancer also had positive anti-PBP serology, preventing its use in discriminating AIP from cancer [28].

Many other antibodies have been associated with AIP but are not diagnostic and include antibodies to carbonic anhydrase II antigens and lactoferrin [30, 31], as well as rheumatoid factor, antinuclear antibody, and anti-smooth muscle antibody [32].

## OTHER MANIFESTATIONS

Extrapancreatic manifestations are found in a significant number of patients with both Type 1 and Type 2 AIP. These have been reported in up to 45% of patients and the prevalence of extrapancreatic involvement in AIP is likely to increase with enhanced awareness of this disease [10]. The most common extrapancreatic site of involvement is the biliary tree, in both Type 1 and Type 2 AIP. In primarily Type 1 disease, other affected organs include salivary glands, chest (including mediastinal fibrosis and adenopathy), retroperitoneum (chronic periaortitis, idiopathic retroperitoneal fibrosis), kidneys (tubulointerstitial nephritis) and orbits (IgG4-associated pseudolymphoma) [4]. IgG4-predominant lymphoplasmacytic infiltrate is often found in affected organs. IgG4-related disease has also been reported in the meninges, aorta, prostate, breast, thyroid, pericardium and skin [4].

Involvement of the biliary tree in autoimmune pancreatitis, sometimes called IgG4 sclerosing cholangitis (IgG4-SC) or IgG4-associated cholangitis (IAC), can be confused with primary sclerosing cholangitis (PSC). Cholangiograms may be able to distinguish between these two entities by highlighting the short band-like biliary strictures, with diverticulum formation and a beaded appearance typical of PSC, compared with the longer, segmental strictures with pre-stenotic dilation found in IgG4-SC [33]. Strictures of the distal common bile duct are also more common in IgG4-SC than in PSC [33].

## PATHOPHYSIOLOGY

AIP is a fibro-inflammatory disorder characterized by a lymphoplasmacytic infiltrate [12]. There is a clear association with auto-antibodies but the exact causative mechanism is yet to be fully elucidated [34, 35]. Several associations with mediators of immunity have been identified. In the Japanese population, there is an increased risk of AIP in patients with the HLA DRB10405-DQB10401 [36]; this was not found in a subsequent study of Korean patients [37]. Cytotoxic T lymphocyte-associated antigen 4 (CTLA-4), which negatively regulates T-cell responses, has been implicated in AIP in both Taiwanese and Japanese patients [38, 39]. However, these HLA and non-HLA genes have not yet been explored in the North American population with AIP.

A variety of auto-antigens have also been suggested in AIP, including antilactoferrin and anticarbonic anhydrase II or -IV [30, 40]. Antibodies to pancreatic secretory trypsin inhibitor and an amino acid sequence of plasminogen-binding protein (anti-PBP) have also been implicated [41].

In a review of the basic science of autoimmune pancreatitis, Park *et al.* [15] discuss animal models and human studies of AIP, highlighting the potential role of cellular immunity [34], the complement system [42], molecular mimickry [43] and regulatory T- cells [44]. Based on this, they have proposed a plausible model for the pathogenesis of AIP that includes an explanation of both Types 1 and 2 AIP.

The role of IgG4 in the pathogenesis of AIP is unknown. IgG4 accounts for the minority of total IgG in an individual. Uniquely, it can participate in half-antibody exchange reactions, where the heavy chain of one IgG4 molecule is replaced by another [45]. This is facilitated by weak, non-covalent forces between the heavy chains of IgG4. The resulting bispecific—but functionally monovalent—IgG4 is unable to bind antigens.

Consequently, IgG4 antibodies have been considered benign and some studies have suggested that they may be protective [46–48]. However, specific IgG4 antibodies have been strongly implicated in mediating diseases such as pemphigus foliaceus [49, 50] and some cases of membranous glomerulonephritis [51, 52]. In AIP, it remains to be elucidated whether IgG4 is a disease-specific driver of inflammation and end-organ damage or, effectively, a bystander produced in response to the inflammatory cascade.

## DISTINGUISHING AIP FROM PANCREATIC CA

An increasingly common scenario in medical practice is distinguishing autoimmune pancreatitis from pancreatic cancer. It is important to recognize that, compared with pancreatic cancer, AIP in the North American population is rare; only 2–3% of patients were incorrectly diagnosed with pancreatic cancer prior to widespread recognition of AIP as a distinct clinical entity [9]. One approach uses an algorithm, based initially on cross-sectional imaging, to stratify patients into three groups: suggestive of cancer, highly suggestive of AIP and supportive of AIP [5, 19]. In patients with imaging supportive of AIP, corroborating evidence is sought through serology for IgG4 and a thorough search for extrapancreatic manifestations. This may allow about 70% of AIP patients to be diagnosed accurately at this stage [19]. In the remaining patients, further evaluation by ERCP with ampullary biopsy or EUS-guided core biopsy can be considered [53, 54]. A steroid trial is an option in carefully selected patients, given the potential for delaying the alternative diagnosis of pancreatic cancer.

## TREATMENT

Unlike other forms of pancreatitis, AIP is very responsive to steroid therapy, therefore making therapy a component of the diagnostic criteria discussed above. Treatment with steroids has been established in many observational trials of heterogeneous patient populations [55–59]. While these results must be interpreted in the context of evolving diagnostic criteria and different definitions of response, the vast majority of both Type 1 and Type 2 AIP patients have clinical improvement with corticosteroid therapy. The response to therapy is determined primarily by resolution of abnormal imaging and extrapancreatic manifestations if initially present, as well as improvement in clinical and biochemical parameters. In a case series at the University of Pittsburgh in 26 patients over 10 years and in a retrospective study of 563 patients in Japan [8, 58], 98–100% responded typically within 12 weeks, with incomplete response in 21%.

There is a variation in treatment patterns within and outside the United States [59]. One commonly used regimen includes treatment with 40 mg of prednisone for four weeks, followed by a taper by 5 mg each week for a total of an 11-week course [5]. Response usually occurs within two to four months. Moon *et al.* have suggested that two weeks may be sufficient to determine response, which may be of particular importance in differentiating AIP from pancreatic cancer without delay [56].

The relapse rate in patients with Type 1 AIP ranges from 30–50% [10, 11], while patients with Type 2 AIP typically do not relapse. Sah *et al.* have identified proximal bile duct involvement and diffuse swelling of the pancreas as risk factors for disease relapse [11]. There remains some debate as to the ability of elevations in serum IgG4 levels to predict relapse. Patients who relapse are typically treated with a second course of corticosteroids [59]. Given the relatively high relapse rates, some centers routinely continue maintenance corticosteroid therapy for up to three years [58]. Kamisawa *et al.* have shown that this reduces relapse rates from 34 to 23%. Others advocate close monitoring and recurrent use of corticosteroids as needed, to minimize therapeutic side-effects. Further, the steroid-sparing immunomodulator, azathioprine, can be used to maintain remission after the first or second relapse. Pannala *et al.* reported that 30–40% of AIP patients will eventually need maintenance therapy to prevent recurrent relapses [59].

In patients refractory to steroids, azathioprine, mycophenolate mofetil, cyclophosphamide and rituximab have all been tried in addition to, or instead of, steroid therapy [60–62]. However, data for their efficacy is limited with the relevant literature primarily consisting of case series and case reports.

## PROGNOSIS

As is expected in the context of an emerging disease entity, there is limited long-term follow-up of patients with AIP. Recent studies have highlighted an increased risk of malignancy in patients with IgG4-related disease. In a study of 106 Japanese patients, 11 were diagnosed with cancer during an average follow-up of 3.1 years [63]. These malignancies included colon cancer, lung cancer and lymphoma and occurred at a frequency about 3.5 times greater than the general population**.** In another study in Japan, 108 patients with AIP were followed for a median of 3.3 years. Almost 15% developed cancer, with the highest risk occurring within the first year of diagnosis of AIP [64]. Some studies have also suggested a slightly increased risk of pancreatic cancer [58, 65].

Despite the increased risk of malignancy, studies have not yet found an effect on mortality. In particular, Sah *et al.* found that long-term survival in patients with either Type 1 or Type 2 AIP was similar to age and gender-matched controls [11].

## CONCLUSIONS

In summary, AIP is a rare fibro-inflammatory disease of the pancreas that is very responsive to steroid therapy. It can be classified into two subtypes, with Type 1 AIP more commonly associated with extrapancreatic manifestations as part of an emerging IgG4 systemic disease. In contrast, Type 2 AIP has a distinct histological profile with a paucity of IgG4 cells and with a low relapse rate. It is important to be aware of this disease entity and to consider it in the differential diagnosis of cholangiocarcinoma, PSC and pancreatic cancer.

**Conflict of interest:** none declared.
